# Complete Genome Sequence of the Heavy-Metal-Tolerant Endophytic Type Strain of *Salinicola tamaricis*

**DOI:** 10.1128/genomeA.00358-18

**Published:** 2018-04-19

**Authors:** Nianjie Shang, Qiaoqiao Zhu, Meixue Dai, Guoyan Zhao

**Affiliations:** a College of Life Science, Shandong Normal University, Jinan, People’s Republic of China; b Shandong Provincial Key Laboratory of Plant Stress Research, Jinan, People’s Republic of China

## Abstract

The first complete genome sequence of a recently described Salinicola tamaricis species was determined for the strain F01^T^ (=CCTCC AB 2015304^T^ =KCTC 42855^T^). The strain was isolated from the leaves of wetland plant Tamarix chinensis Lour and shows a high tolerance to heavy metals, such as manganese, nickel, lead, and copper ions.

## GENOME ANNOUNCEMENT

Wetlands have been proposed as sites for sinks of metal contaminants. The heavy metals can be taken up, transported, and released by wetland plants, known as the process of phytoremediation (1). In past decades, a combination of endophytic bacteria and their plant hosts for the increased remediation of pollutants has been applied successfully for the removal of toxic metals from contaminated soils (2, 3). Moreover, many metal-resistant endophytes have been reported to promote plant growth by mechanisms, such as solubilization of minerals and production of phytohormones and siderophores (2). We have isolated several heavy-metal-resistant strains from the leaves of halophytes collected from the saline soil along Yellow River Delta, China. Among these isolates, strain F01^T^ from Tamarix chinensis Lour was recognized as a new Salinicola tamaricis strain in early 2017. It is the type strain of S. tamaricis and was assigned the name strain F01 (=CCTCC AB 2015304^T^ =KCTC 42855^T^) (4). Strain F01^T^ was able to grow in culture media with high concentrations of heavy-metal ions, such as Mn^2+^, Fe^2+^, Cu^2+^, Pb^2+^, Ni^2+^, Cd^2+^, and Zn^2+^ (4). Here, we report the complete genome sequence of S. tamaricis.

A total of 211,466 PacBio raw reads were filtered, as per the read qualities. The cleaned reads were then assembled using SOAP*denovo* software version 2.04 (5). The assembly results were evaluated based on the distribution of k-mer frequency, GC content, and genome coverage depth. This yielded a 4,276,240-bp genome sequence composed of 1 scaffold and 1 contig, with 65.68% GC content. The coding sequences were predicted by using Glimmer version 3.02 (6). Functional annotation was achieved using databases, including Gene Ontology (GO) (7), the Kyoto Encyclopedia of Genes and Genomes (KEGG) (8, 9), Swiss-Prot, the Cluster of Orthologous Groups (COG) (10), and the Nonredundant (NR) protein databases. Noncoding genes and miscellaneous features were predicted using the RNAmmer (11), tRNAscan-SE (12), and Rfam (13) softwares. A total of 4,724 coding sequences and 120 RNAs, 72 tRNAs, and 15 rRNAs were predicted.

Additionally, the genomic data of S. tamaricis strain F01^T^ showed about 50 genes involved in resistance to antibiotics, such as macrolide (at positions 442990 to 443721 bp in GenBank accession no. CP023559), penicillin (at positions 340058 to 340648 bp in GenBank accession no. CP023559), and trimethoprim (at positions 367805 to 368362 bp in GenBank accession no. CP023559), indicating that high adaptational abilities of strain F01^T^ have been developed toward stressful environmental conditions.

Taken together, the complete genome sequence of strain F01^T^ was obtained. With further analysis in the future, these data will serve as a baseline for the molecular studying of detoxification mechanisms of endophytic bacteria during the phytoremediation process of the plant-soil-microbe system.

### Accession number(s).

The whole-genome sequence of Salinicola tamaricis strain F01^T^ has been deposited in DDBJ/ENA/GenBank under the accession no. CP023559. The version described in this paper is the first version, CP023559.1.
